# Stable and Variable Parts of Microbial Community in Siberian Deep Subsurface Thermal Aquifer System Revealed in a Long-Term Monitoring Study

**DOI:** 10.3389/fmicb.2016.02101

**Published:** 2016-12-27

**Authors:** Yulia A. Frank, Vitaly V. Kadnikov, Sergey N. Gavrilov, David Banks, Anna L. Gerasimchuk, Olga A. Podosokorskaya, Alexander Y. Merkel, Nikolai A. Chernyh, Andrey V. Mardanov, Nikolai V. Ravin, Olga V. Karnachuk, Elizaveta A. Bonch-Osmolovskaya

**Affiliations:** ^1^Department of Plant Physiology and Biotechnology, Tomsk State UniversityTomsk, Russia; ^2^Federal Research Centre (FRC) Biotechnology, Institute of BioengineeringMoscow, Russia; ^3^Federal Research Centre (FRC) Biotechnology, Winogradsky Institute of Microbiology, Research Center of Biotechnology, Russian Academy of Sciences (RAS)Moscow, Russia; ^4^Glasgow and Holymoor Consultancy Ltd., Glasgow UniversityChesterfield, UK

**Keywords:** deep subsurface, thermophilic microbial communities, element cycles and biogeochemical processes, Western Siberia, phylogenetic analysis

## Abstract

The goal of this work was to study the diversity of microorganisms inhabiting a deep subsurface aquifer system in order to understand their functional roles and interspecies relations formed in the course of buried organic matter degradation. A microbial community of a deep subsurface thermal aquifer in the Tomsk Region, Western Siberia was monitored over the course of 5 years via a 2.7 km deep borehole 3P, drilled down to a Palaeozoic basement. The borehole water discharges with a temperature of ca. 50°C. Its chemical composition varies, but it steadily contains acetate, propionate, and traces of hydrocarbons and gives rise to microbial mats along the surface flow. Community analysis by PCR-DGGE 16S rRNA genes profiling, repeatedly performed within 5 years, revealed several dominating phylotypes consistently found in the borehole water, and highly variable diversity of prokaryotes, brought to the surface with the borehole outflow. The major planktonic components of the microbial community were *Desulfovirgula thermocuniculi* and *Methanothermobacter* spp. The composition of the minor part of the community was unstable, and molecular analysis did not reveal any regularity in its variations, except some predominance of uncultured *Firmicute*s. Batch cultures with complex organic substrates inoculated with water samples were set in order to enrich prokaryotes from the variable part of the community. PCR-DGGE analysis of these enrichments yielded uncultured *Firmicutes, Chloroflexi*, and *Ignavibacteriae*. A continuous-flow microaerophilic enrichment culture with a water sample amended with acetate contained *Hydrogenophilus thermoluteolus*, which was previously detected in the microbial mat developing at the outflow of the borehole. Cultivation results allowed us to assume that variable components of the 3P well community are hydrolytic organotrophs, degrading buried biopolymers, while the constant planktonic components of the community degrade dissolved fermentation products to methane and CO_2_, possibly via interspecies hydrogen transfer. Occasional washout of minor community components capable of oxygen respiration leads to the development of microbial mats at the outflow of the borehole where residual dissolved fermentation products are aerobically oxidized. Long-term community analysis with the combination of molecular and cultivation techniques allowed us to characterize stable and variable parts of the community and propose their environmental roles.

## Introduction

The deep subsurface is one of the largest habitats for prokaryotes, and the total biomass of subsurface microbes probably exceeds the numbers in the rest of the biosphere (Whitman et al., 1998; McMahon and Parnell, 2014). A number of studies have demonstrated sizable and metabolically active subsurface microbial communities in the deep sub-seafloor (Parkes et al., 2000; Kimura et al., 2003; Takai et al., 2004; Teske, 2005; Batzke et al., 2007; Edwards et al., 2011; Lomstein et al., 2012). Abundant and diverse microbial communities have been revealed in terrestrial deep subsurface habitats all over the world (Fredrickson and Hicks, 1987; Ghiorse and Wilson, 1988; Jiménez, 1990; Takai et al., 2001; Itävaara et al., 2011; Bomberg et al., 2015; Frank et al., 2016). Some studies have suggested that microbes in the deep subsurface have extremely slow in-situ growth rates because of the lack of detrital energy inputs over thousands to millions of years (Onstott et al., 2014; Lever et al., 2015). While uncultured and poorly studied microorganisms are speculated to play major roles in geochemical cycles in these environments (Wrighton et al., 2012; Castelle et al., 2013), a great deal of uncertainties exists, stimulating a search for novel microorganisms from subsurface ecosystems. Characterization of microbial and metabolic diversity in the deep terrestrial subsurface is in incipient stages and very far from complete (Hug et al., 2016).

Deep terrestrial subsurface environments may represent extreme habitats with high pressure, temperature, and/or salinity (Ollivier et al., 2007). Depending on the energy source, deep subsurface microbial communities could be “lithoautotrophic” and “organotrophic” (Fredrickson and Hicks, 1987). In the former ones, molecular hydrogen of abiotic origin is considered to be the main source of energy (Takai et al., 2004; Nealson et al., 2005; Pedersen, 2012), while, in the latter, the buried and completely or partly altered organic matter (kerogen or crude oil) provides energy substrates that fuel deep subsurface microbial communities. These communities have been sampled through drilled boreholes penetrating deep strata. In majority, the drilling activity was related to hydrocarbon exploration. Subsurface microbial communities have been sampled from the high-temperature hydrocarbon reservoirs of Western Siberia, Kazakhstan (Nazina et al., 1995; Bonch-Osmolovskaya et al., 2003; Frank et al., 2016), California (Orphan et al., 2003), North Sea (Dahle et al., 2008), China (Li et al., 2006; Nazina et al., 2006), etcetera. Microbes from borehole water samples represent the planktonic community, while microorganisms on mineral surfaces and in biofilms in subsurface environments remain elusive as they only appear in the borehole fluid sporadically (Edwards et al., 2005; Wanger et al., 2006). In some cases, this sporadic distribution of the immobilized microorganisms could lead to erroneous interpretation of subsurface community compositions judged by singular or irregularly repeated molecular fingerprints. To date, only a few research projects have concentrated on the long-term dynamics of deep subsurface communities (e.g., Wu et al., 2016).

Our work is part of a long-term study of microbial communities inhabiting deep thermal aquifers in the Mesozoic and Palaeozoic sedimentary sequences of the Western Siberian megabasin. During the 1950s, many hydrocarbon exploration wells drilled in this region penetrated deep aquifers rather than significant hydrocarbon reservoirs. The borehole depths typically varied from 500 to 4000 m, with water temperatures ranging from 20 to 140°C. In this study, we have characterized the microbial community of the borehole 3P (alternative designations in other publications: P3, P-3, 3-R or “Goryachii Istochnik”) situated on the west bank of the River Ob', between the ports of Parabel' and Narym, and in the Parabel' District, Tomsk Region of the Russian Federation. The borehole was drilled in 1957–1958 and was left unsealed. The thermal water overflowing from the borehole has subsequently been tapped for a spa resort: it discharges uncontaminated to the surface of a wooden conduit above the ground level. On the conduit, non-photosynthetic microbial mats develop downstream of the outflow (Figure S1). The goal of this work was to study the composition of microbial community in water during several years in order to find how stable it is and what the functional roles of its components are. Both borehole water and microbial mats were sampled in this study over a five-year period, from August 2009 to August 2013. Changes in the composition of planktonic microbial communities were traced by PCR-DGGE analysis of the 16S rRNA gene fragments. High-throughput sequencing of variable 16S rRNA gene fragments was additionally performed for a comprehensive analysis of the prokaryotic communities. Previous studies of deep subsurface aquifers showed that only 0.022–1% of all cells are present in water in an unattached state (McMahon and Parnell, 2014). We assumed that hydrolytic microorganisms attached to the organic-rich ore would be present in water as minor components and could be revealed in specific enrichment cultures. Thus, an attempt was made to study the hydrolytic part of a deep subsurface population by batch enrichment cultures further analyzed by PCR-DGGE of 16S rRNA gene fragments. A continuous microaerophilic acetate-utilizing enrichment from a water sample was also established. It was supposed to reproduce the microbial mat developing at the outflow of the 3P well. Therefore, the combination of different approaches allowed us to reveal stable planktonic and variable immobilized parts of the deep subsurface microbial community as well as provide insight into the origin and diversity of microbial mats consuming organic molecules present in deep subsurface water.

## Materials and methods

### Site description and samples collection

Borehole 3P is located on the west bank of the River Ob' (N58°50′, E81°30′) in the Tomsk Region, Western Siberia, Russia. According to different sources, the depth of the borehole is 2609 or 2775 m. It was drilled as an oil exploration well in 1957–1958 and then abandoned and harnessed by the local population as a source of thermal water. The borehole penetrated almost 30 m of Quaternary sediments, followed by 182 m of Palaeogene sediments. This was succeeded by a Cretaceous sedimentary sequence down to 2250 m and a Jurassic sequence to 2600 m. At 2600 m, the borehole reached the Palaeozoic basement, thus comprising the monzonite and granite (Ulmishek, 2003; Banks et al., 2011, 2014). Water discharges at approximately 9 m^3^ day^−1^, has a moderate mineralization (total dissolved solids 13–14 g l^−1^) and contains dissolved H_2_S and CH_4_. Methane exsolves as bubbles on the surface. The stratigraphic location from which the water is derived is not known with certainty. Given a water temperature of around 50°C and a typical geothermal gradient in tectonically stable areas of 20–30°C per km, and allowing for the fact that the water may cool during its ascent along the borehole, it seems likely that the water is derived from near the base of the borehole.

Samples of water were collected seven times: in August 2009, February 2010, March 2010, June 2011, July 2012, September 2012, and August 2013. Temperature, pH, and redox potential of water were measured on-site (HI 8314 Hanna pH/redox-meter and corresponding electrodes). Samples for chemical, molecular, and microbiological analyses were taken from the wellhead into sterile 500 ml serum bottles sealed with rubber stoppers. Stoppers of sample containers for analysis of organic compounds were covered with Teflon films. All samples were kept at +4°C for 24 h before analysis. Gas samples were collected four times: in August 2009, March 2010, June 2011, and July 2012. For the samples with the highest hydrocarbon content (2011), the carbon isotopic composition of the gas phase was determined. Gas bubbles exsolving from thermal water were collected by a method modified from those described by Giggenbach and Goguel (1989). We used a glass gas sampler (Savannah River National Laboratory) connected with a sterile 60 ml bottle by a Norprene tubing and submerged into the tank with thermal water inflow. The gas accumulating in the sampler was collected into a bottle by displacement of 50 ml thermal water from it, and 10 ml of water were left as a hydroseal. The filled bottles, being held under water, were sealed with butyl rubber stoppers and then capped and transferred to the laboratory upside down to prevent post-sampling atmospheric contamination. Samples of microbial mats growing along the water flow were collected three times (in August 2009, February 2010, and March 2010) in 50 ml Falcon tubes and were stored at 4°C until used.

### Hydrochemical analyses

Water samples were filtered (0.22 μm) using Millex-GS filter units (Merck Millipore) before analysis. Organic compounds were extracted with chloroform from the water samples. Liquid chromatography–mass spectrometry (LC-MS) was performed in the Institute of Petroleum Chemistry, Siberian branch of Russian Academy of Sciences (Tomsk, Russia). Determination of selected organic compounds was carried out using a Thermo Scientific DFS mass spectrometer. Major ions and chemical oxygen demand (COD) were determined by the Scientific Educational Production Center Voda of Tomsk Polytechnic University by titration (bicarbonate and chloride) or spectrophotometry (sulfate), and H_2_S was measured colorimetrically with *N,N*-dimethyl-*p*-phenylenediamine (dihydrochloride salt) as the chromophore (Cline, 1969). For COD measurements, the sample was refluxed with potassium dichromate in the sulfuric acid medium and the excess potassium dichromate was determined by titration against ferrous ammonium sulfate using ferroin as an indicator. Other ions were determined at the Chemical Analytical Center Plasma (Tomsk) using an ICP mass-spectrometer ELAN model DRC-e (PerkinElmer Instruments).

In 2010, parallel samples (0.45 μm filtration) were analyzed by ICP-AES and ion chromatography at the Geological Survey of Norway (Banks et al., 2011). In 2013, parallel samples (0.45 μm filtration) were analyzed by ICP-MS and ion chromatography at the British Geological Survey.

Gas samples were analyzed using the Crystal 5000.1 gas chromatograph (Chromatek) equipped with a HayeSep N 80–100 mesh Supelco column (Sigma-Aldrich), methanator, FID, and TCD detectors, which were conditioned at 40°, 350°, 200°, and 150°C, respectively. The carrier gas was argon (25 ml min^−1^). Volatile fatty acids in the water samples were analyzed using the same equipment with a FID detector and Porapak column conditioned at 180°C. The isotopic composition of methane and ethane (δ^13^C) was determined on a Thermo Electron gas chromatograph coupled with a Delta plus mass spectrometer (Thermo Fisher Scientific).

### DNA extraction

DNA was extracted from water and mat samples, enrichment cultures, and isolates. Cells from 1.5 l aliquots of borehole groundwater were retained on cellulose nitrate filters (0.2 μm) using a Sartorius filtration unit. Filters were pestled in liquid nitrogen at −70°C and then used for DNA extraction with a Power Soil DNA isolation kit (MO BIO Laboratories) according to the manufacturer's instructions. Cells from enrichment culture broths and pure cultures were collected by centrifugation (9000 g for 15 min). Extraction of DNA from approximately 5 ml of enrichments or pure cultures was performed as described by Tsai and Olson (1991), with minor modifications (i.e., proteinase K (10 μg ml^−1^) was added after three cycles of freezing the sample in liquid nitrogen at −70°C and thawing in a 65°C water bath).

### PCR amplification

Nested PCR (Vissers et al., 2009) was used for the amplification of bacterial and archaeal 16S rRNA genes with the primers listed in Table S2. Domain-specific primers were used separately for Bacteria and Archaea for both the first and second rounds of PCR. The products obtained in the reaction with outer primers were diluted with nuclease-free water (Fermentas) to a concentration of ca. 100 mg l^−1^ and then used as a template for the reaction with inner primers. 16S rRNA gene fragments of Bacteria were first amplified using outer primer pair 27F–1492R (DeLong, 1992) and then with Bacteria-specific inner primer pair GC-Bacv3f and 907r (Lane, 1991; Muyzer et al., 1996). The first round of amplification of the 16S rRNA gene fragments of Archaea was conducted with the primer pair 21F-958R (Weisburg et al., 1991; DeLong, 1992) and the second round with the Archaea-specific pair GC-Arch915r and Parch519f (Coolen et al., 2004). We have successfully used these primer sets in our previous studies to amplify bacterial and archaeal 16S rRNA gene fragments separately (Karnachuk et al., 2009; Frank et al., 2016).

Table S2 shows the details of the PCR conditions used in this study. The amounts of MgCl_2_, dNTPs, primers, and Taq DNA-polymerase were altered depending on the primer set. The reagents used were 25 mM MgCl_2_, 2 M dNTPs, 5 U μl^−1^ *Taq* DNA polymerase, 10x *Taq* buffer, and nuclease-free water (Fermentas). 100 μM stock solutions of oligonucleotide primers synthesized by Syntol (Moscow) were applied. 400 mg l^−1^ of BSA (Fermentas) were added to the reaction mixture for archaeal 16S rRNA gene amplification. Reactions with outer primers were conducted in a volume of 50 μl. PCR with inner primers was performed in a volume of 100 μl. Each mixture contained 50–100 mg l^−1^ of the DNA matrix. All PCRs were conducted in the MyCycler thermal cycler (Bio-Rad Laboratories).

### Denaturing gradient gel electrophoresis (DGGE)

The DGGE used for the separation of amplified fragments (Muyzer et al., 1993) was performed with the DCode System (Bio-Rad). Polyacrylamide gel (8%) with a 30–70% denaturing gradient was used for 16S rRNA fragments separation (100% denaturing solution contained 7 M urea and 40% formamide). Electrophoresis was performed for 17 h at 60°C and 120 V. For separation of archaeal 16S rRNA gene fragments, 8% polyacrylamide gel with 20 to 80% denaturing gradient was used. Electrophoresis was performed for 19.5 h at 60°C and 100 V, as described by Vissers et al. (2009). DGGE gel was stained with 0.5 mg l^−1^ ethidium bromide (Bio-Rad) in a TAE buffer for 15 min and then washed in TAE for 20 min. The stained gel was visualized in UV light (320 nm) using a GelDoc-It imaging system (UVP, UK). Separate bands were cut from the gel in UV (320 nm) using transilluminator ECX-26MX (Vilber Lourmat). Then DNA was extracted to 20 μl of nuclease-free water (Fermentas) for 12 h at 4°C. Amplification of 16S rRNA gene fragments was performed as described above, but the primers did not contain GC-clamps. DGGE analysis of each PCR product was performed in duplicate.

### Phylogenetic analysis

Commercial sequencing of 16S rRNA (585 bp and ca. 400 bp) gene fragments was performed by Syntol. The sequences were analyzed against the GenBank nucleotide collection database (http://www.ncbi.nlm.nih.gov/blast) by the BLASTN algorithm with standard parameters (Altschul et al., 1997). The sequences were aligned using BioEdit sequence alignment editor (Hall, 1999), Version 7.2.5. Maximum likelihood and neighbor-joining phylogenetic trees based on the comparison of 16S rRNA gene sequences were constructed using MEGA 6 (Tamura et al., 2013). All the sequences were uploaded to the GenBank nucleotide collection database under the accession numbers KT897597 – 651, KY010808, and KY010809.

### Pyrosequencing of 16S rRNA gene fragments

Pyrosequencing analyses were performed for water samples obtained from the borehole in June 2011. Cells from 30 L of water were collected on 0.2 μm nitrate cellulose filters. The filters were frozen in liquid nitrogen and then ground and melted with TE buffer in a water bath at 37°C. The total DNA was extracted by the CTAB/NaCl method (Wilson, 2001).

Universal primers were used for amplification of the V3–V4 variable regions of the 16S rRNA gene, U341F (5′-CCT ACG GGR SGC AGC AG-3′), and PRK806R (5′-GGA CTA CYV GGG TAT CTA AT-3′). The primer check with SILVA TestPrime confirmed that they target most of Archaea and Bacteria except only a few lineages, notably Planctomycetes and some Crenarchaeota. The PCR fragment was pyrosequenced on GS FLX (Roche) using titanium chemistry. A total of 39,276 sequence reads were obtained. Most of the reads covered the full length of the PCR fragment. The reads with mismatches to primer sequences, those containing ambiguous nucleotides, and those shorter than 400 bp were excluded from the analysis. Then 16S rRNA data were analyzed by the RDP Classifier program package (Cole et al., 2009). Initially, the sequences obtained with universal primers were distributed between Bacteria and Archaea using the online RDP Naive Bayesian rRNA Classifier Version 2.0 (http://rdp.cme.msu.edu/classifier/classifier.jsp). Subsequently, bacterial and archaeal 16S rRNA gene datasets were analyzed separately and subjected to additional filters. First, AmpliconNoise (Quince et al., 2011) was used to account for homopolymer-derived and PCR errors. Then all remaining singletons (unique sequences occurring only once) were removed, as suggested by Behnke et al. (2011). The final datasets consisted of 21,608 bacterial and 6965 archaeal 16S reads. Complete linkage clustering and selection of representative sequences for operational taxonomic units (OTUs) were performed using the RDP Classifier (Cole et al., 2009). OTUs were assigned to taxonomic groups (i.e., bacterial and archaeal divisions) on the basis of BLASTN sequence similarity searches against the NCBI database. Taxonomic assignments were refined following the construction of phylogenetic trees consisting of representative sequences of the clusters and a set of 16S rRNA gene sequences from the representatives of different archaeal and bacterial lineages. The sequences were aligned using CLUSTALX (Thompson et al., 1997), and the neighbor-joining tree was computed by TREECON (Van de Peer and De Wachter, 1994). Shannon and equitability indices based on 16S rRNA gene sequence data were calculated at the 97 and 95% cutoff levels using the RDP Pipeline (Cole et al., 2009). Pyrosequencing read data has been deposited in the NCBI SRA database under accession SRR4450631.

### Enrichment of deep subsurface microorganisms in batch and continuous cultures and isolation of pure cultures

Microorganisms of different metabolic groups were enriched using aerobic and anaerobic conditions as previously described (Podosokorskaya et al., 2013a). Microcrystalline cellulose (Avicel; 2 g l^−1^), cellobiose (2 g l^−1^), lactate (Na salt; 20 mM), peptone (Sigma; 10 g l^−1^), gelatin (Sigma; 20 g l^−1^), and H_2_ (80 or 100%) were used as the energy substrates. Sulfate (Na salt, 10 mM), arsenate (Na salt, 5 mM), and glauconite (final Fe(III) concentration ca. 20 mM) were employed as the electron acceptors. Yeast extract (0.05–0.1 g l^−1^) was added as the source of growth factors. For batch enrichments, 10 ml portions of the media were dispensed in 18 ml Hungate tubes, and the head space was filled with an oxygen-free N_2_(100%) or N_2_/CO_2_ mixture (80:20%). When the medium was prepared aerobically or H_2_ was used as the substrate, the volume of the medium was 5 ml, and the head space was filled with either air or H_2_ (100%) or H_2_/CO_2_ mixture (80:20%), respectively. Incubation temperatures were 47, 50, 54, or 70°C. The growth of all cultures was monitored by direct cell counting under a phase contrast microscope at 1000 × magnification.

Continuous enrichments were initiated in a 1.3 l BioFlo 110 bioreactor (New Brunswick Scientific) under microaerobic conditions (4.5% O_2_ in the gas phase) with Na-acetate (10 mM) as the substrate. The mineral salts solution was modified according to the chemical analysis results of the borehole water as follows. The mineral salts solution contained (per liter) 7.0 g NaCl, 0.19 g MgCl_2_, 0.25 g NH_4_Cl, 0.5 g KCl, 0.2 g KH_2_PO_4_, 0.15 g NaHCO_3_, 0.013 g Na_2_S, and 1.0 g CaCl_2_. The medium was prepared as described previously (Podosokorskaya et al., 2013a). Trace element solutions A (10 ml l^−1^) and B (1 ml l^−1^) (Table S3) were used to provide for minor and trace elements corresponding to the elemental composition of the borehole water. Spherical glass beads (av. diameter 2 mm) were spread at the bottom of the reactor before autoclaving to model the mineral surface of the wallrock in the aquifer. The medium was autoclaved in the bioreactor and feed bottles at 121°C for 20 min. The pH (7.0–7.5), temperature (50–70°C), and dissolved O_2_ of the medium were adjusted before inoculation and automatically sustained during the incubation. The bioreactor was inoculated with 0.5 l of borehole water sampled in February 2010 and started in a batch mode. At the commencement of the exponential growth phase, the bioreactor was switched to continuous mode at a gradually increasing dilution rate of 0.5–1.17 ml min^−1^. Mixing was adjusted with Rushton impellers to 160 ml min^−1^ (coefficient of turbulent diffusion 178.2 cm^2^ s^−1^), which ensured a perfect mixing model. The composition of enrichment cultures was studied using PCR-DGGE analyses as described above.

## Results

### Inorganic hydrochemistry of the borehole water

The chemical analysis results of the borehole water, sampled at three different time points in 2009–2010, have been reviewed by Banks et al. (2011), and the sampling in August 2013 has also been summarized (Banks et al., 2014). Briefly, the water is of Na-(Ca)-Cl composition. The molar ratio of Na/Cl is between 0.78 and 0.86, which is almost identical to modern sea water (0.86). Bromide occurs at 28–33 mg l^−1^ in the water, while the Cl^−^/Br^−^ mass ratio of around 262–275 is very close to 288 of standard seawater. The water contains ca. 1.1 g l^−1^ of Ca but only about 5 mg l^−1^ of Mg. It is supersaturated with respect to calcite and saturated with respect to dolomite; the Sr concentration is high at ca. 86 mg l^−1^.

A significant variation in temperature was observed, from 50.2°C in February 2010 to 45.2°C in August 2010 (Table 1), while the temperature was around 50°C in August 2013. The water pH was circumneutral and also varied somewhat from 7.52 in August 2009 to 6.91 in August 2010. The redox potential of about −250 to −300 mV (vs. standard hydrogen electrode [SHE]) was indicative of strongly reducing conditions, also evidenced by the presence of dissolved H_2_S, CH_4_, near absence of sulfate, and elevated barium (17 mg l^−1^ in 2013) due to the lack of a barite solubility ceiling. The H_2_S concentrations varied from 2.46 mg l^−1^ in August 2009 to 7.42 mg l^−1^ in August 2010. Concentrations of most heavy metals (except Ba) measured in 2013 were low and were presumed to be suppressed by the high pH and sulfide concentrations. Arsenic concentrations were ca. 130 μg l^−1^ (Table S1).

**Table 1:** **Characteristics of 3P borehole water determined at four different sampling times**.

**Parameter**	**August 2009**	**February 2010**	**August 2010**	**August 2013**
T°C	46.9	50.2	45.2	50.0
pH	7.5	7.4	6.9	7.3
E_h_, mV (vs SHE)	−248	−299	−284	−283
Salinity, mg l^−1^	15000	15000	13500	n.m.
COD, mg O_2_ l^−1^	4400	201	177	n.m.
${\text{SO}}_{4}^{2-}$, mg l^−1^	4.2	<2.0	<2.0	25.0
${\text{NH}}_{4}^{+}$, mg l^−1^	12.9	6.6	5.7	n.m.
HCO_3_−, mg l^−1^	182.0	140.3	135.0	183.0
H_2_S, mg l^−1^	2.4	3.2	7.4	n.m.
Alkanes C_10_-C_20_, μg l^−1^	5.0	2.5	12.7	n.m.
Alkanes C_21_-C_35_, μg l^−1^	3.1	6.4	7.5	n.m.
C_6_-C_16_ carboxylic acids, μg l^−1^	3.8	0.4	7.5	n.m.

*n.m., not measured*.

### Organic hydrochemistry of the borehole water

The concentration of total carbon, including organics (as judged by COD measurements), varied significantly from ca. 10^3^ mg l^−1^ in August 2009 to ca. 10^2^ mg l^−1^ in 2010 sampling rounds. Modest concentrations of alkanes and non-volatile C_6_–C_16_ carboxylic acids were detected in all samples (Table 1). Acetate (160 mg l^−1^) and propionate (7.5 mg l^−1^) were present in significant concentrations, which underwent minor changes in the scope of 18% from August 2009 to March 2010. No other volatile fatty acids were detected in water samples.

### Gas composition of the borehole water

The composition of the exsolved gas bubbles collected from the water varied with time. CH_4_ was the main constituent, varying from 57.5% (v/v) (June 2011) to 86.3% (March 2010). The N_2_ content reached up to 21.6% of the gas in June 2011 (Table 2). O_2_ was detected in almost all gas samples, probably originating from nonequilibrium degassing of admixed meteoric recharge upon contact with deep hot sedimentary water. The minor components detected in almost all the samples were CO_2_ and H_2_ with concentrations not exceeding 0.3%. An ethane concentration of 16.3% was recorded in the June 2011 sample when methane was at its lowest fraction, but it decreased to 0.8% in the July 2012 sample (Table 2). The isotopic composition (δ^13^C) of CH_4_ and CH_3_CH_3_ in the June 2011 sample was −53%0 (indicative of thermogenic with minor admixture of biogenic CH_4_ as described by Schoell, 1984) and −40%0, respectively.

**Table 2:** **Composition of gas mixture of 3P borehole**.

**Component**	**Concentration, % (v/v) in the samples by month/year**			
	**August 2009**	**March 2010**	**June 2011**	**July 2012**
CH_4_	74.7	86.3	57.5^b^	75.8
N_2_	21.0	13.3	21.6	13.5
Ethane with admixtures^a^	Nd	Nd	16.3^b^	0.8
O_2_	4.0	0.2	4.5	1.8
CO_2_	0.3	Nd	0.1	0.2
H_2_	0.01	0.09	Nd	Nd

a *For the June 2011sample, ethane comprised up to 90% of gaseous hydrocarbons (excluding methane), and other components of this mixture have not been analyzed; in July 2012, the mixture of gaseous hydrocarbons comprised 83% ethane, 13% propane, and 4% n- and iso-butane (total)*.

b *The isotopic composition (δ^13^C) has been determined (refer to the text)*.

*Nd, not detected*.

### Microbial cell numbers and morphotypes in the borehole water

Cell numbers assessed by direct cell counting varied significantly from one sample to another, being maximal in the first water sample taken in August 2009 (ca. 10^8^ cells ml^−1^) and minimal in the February 2010 sample (3 × 10^4^ cells ml^−1^). In other water samples, the cell content analyzed on-site varied from 10^5^ to 10^7^ cells ml^−1^. In the sample, taken in June 2011 for 16S rRNA-profiling with the pyrosequencing technique, the cell number comprised ca. 2 × 10^5^ cells ml^−1^. In all the samples, various short rod-shaped morphotypes (less than 1 μm in length) prevailed and two different coccoid morphotypes were also detected.

### PCR-DGGE analysis of archaeal and bacterial diversity in the borehole water

**Figure 1:**
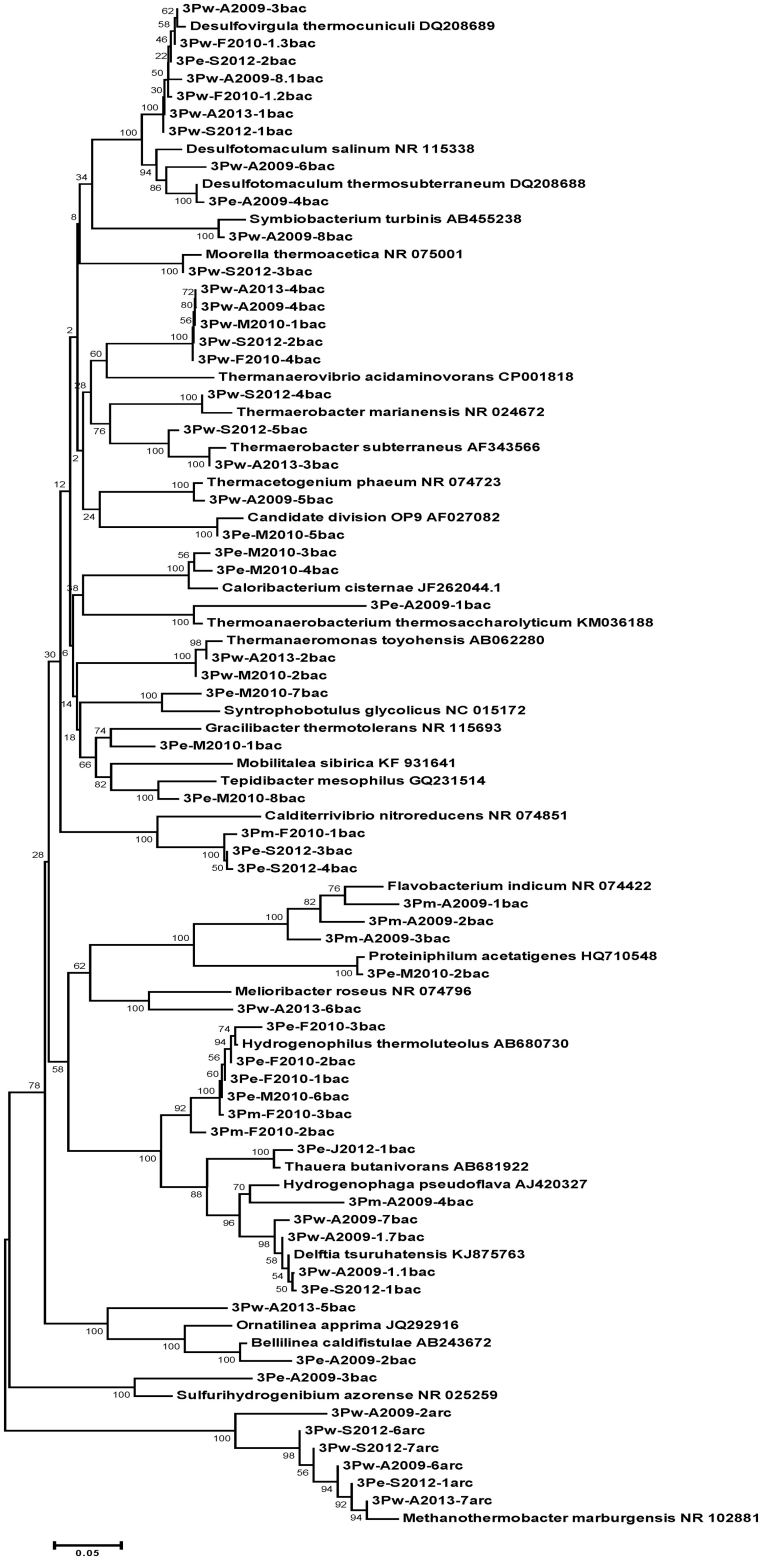
**Phylogenetic position of Bacteria and Archaea detected in water, mats, and enrichments of 3P well by PCR-DGGE analysis**. 3P, general designation; w, water; m, mat; e, enrichment; M2010, month and year of sampling; N, number of sequence; bac, bacteria; arc, archaea. Bootstrap values based on 1000 replications are shown at branch nodes.

Some members of the prokaryotic community matched with bacterial genera (95–100% similarity), namely *Delftia, Desulfovirgula, Thermoacetogenium, Desulfotomaculum, Symbiobacterium, Ignavibacterium*, and archaea in the genus *Methanothermobacter*. Many phylotypes in the borehole water were uncultured *Firmicutes* (88–89% 16S rRNA gene similarity with closest cultured relatives). The majority of phylotypes were detected only once or, occasionally, twice. Among these, there were phylotypes matched with *Delftia tsuruhatensis* (only August 2009) and *Ignavibacterium album* (March 2010 and August 2013). Three phylotypes from the August 2009 sample were consistent with *Thermoacetogenium phaeum* (99% similarity), *Symbiobacterium turbinis* (97%), and *Desulfotomaculum salinum* (99%). Two *Firmicutes* were present in four out of the five DNA samples: a sulfate-reducing thermophile *Desulfovirgula thermocuniculi* (98–99%) and an uncultured bacterium that had 86% similarity with *Thermanaerovibrio acidaminovorans* (Table 3, Figure 1).

**Table 3:** **Prokaryotic diversity in 3P borehole water and the microbial mat developing at the borehole outflow according to PCR-DGGE analyses of 16S rRNA genes**.

**Designation of 16S rRNA sequence**	**Closest cultured relative, % of 16S rRNA gene identity**	**August 2009**	**February 2010**	**March 2010**	**September 2012**	**August 2013**
**WATER SAMPLES**						
3Pw-A2009-1.1bac	*Delftia tsuruhatensis*, 99%	+				
3Pw-A2009-7bac		+				
3Pw-A2009-1.7bac		+				
3Pw-A2013-1bac	*Desulfovirgula thermocuniculi*, 98–99%					+
3Pw-S2012-1bac					+	
3Pw-F2010-1.2bac			+			
3Pw-F2010-1.3bac			+			
3Pw-A2009-8.1bac		+				
3Pw-A-2009-3bac		+				
3Pw-A2013-4bac	*Thermanaerovibrio acidaminovorans*, 87%					+
3Pw-A2009-4bac		+				
3Pw-F2010-4bac			+			
3Pw-S2012-2bac					+	
3Pw-M2010-1bac				+		
3Pw-M2010-2bac	*Thermaeromonas toyohensis*, 88%			+		
3Pw-A2013-2bac						
						+
3Pw-A2009-5bac	*Thermoacetogenium phaeum*, 99%	+				
3Pw-A2009-6bac	*Desulfotomaculum salinum*, 99%	+				
3Pw-A2009-8bac	*Symbiobacterium turbinis*, 97%	+				
3Pw-S2012-3bac	*Moorella thermoacetica*, 88%				+	
3Pw-S2012-4bac	*Thermaerobacter marianensis*, 88%				+	
3Pw-S2012-5bac	*Thermaerobacter subterraneous*, 89%				+	
3Pw-A2013-3bac						
						+
3Pw-M2010-3bac	*Ignavibacterium album*, 98%			+		
3Pw-A2013-6bac						
						+
3Pw-A2013-5bac	*Sphaerobacter thermophilus*, 83%					+
3Pw-A2009-2arc	*Methanothermobacter marburgensis*, 95–99%	+				
3Pw-F2010-2arc			+			
3Pw-S2012-6arc					+	
3Pw-A2009-6arc		+				
3Pw-A2013-7arc						+
**MICROBIAL MAT SAMPLES**						
3Pm-A2009-1bac	*Flavobacterium kamogawaensis*, 94%	+				
3Pm-A2009-2bac	*Flavobacterium cucumis*, 94%	+				
3Pm-A2009-3bac	*Flavobacterium gelidilacus*, 94%	+				
3Pm-A2009-4bac	*Hydrogenophaga pseudoflava*, 95%	+				
3Pm-F2010-2bac			+			
3Pm-F2010-3bac	*Hydrogenophilus thermoluteolus*, 96-99%		+			

### Pyrosequencing analysis of 16S rRNA gene fragments in the borehole water

**Figure 2:**
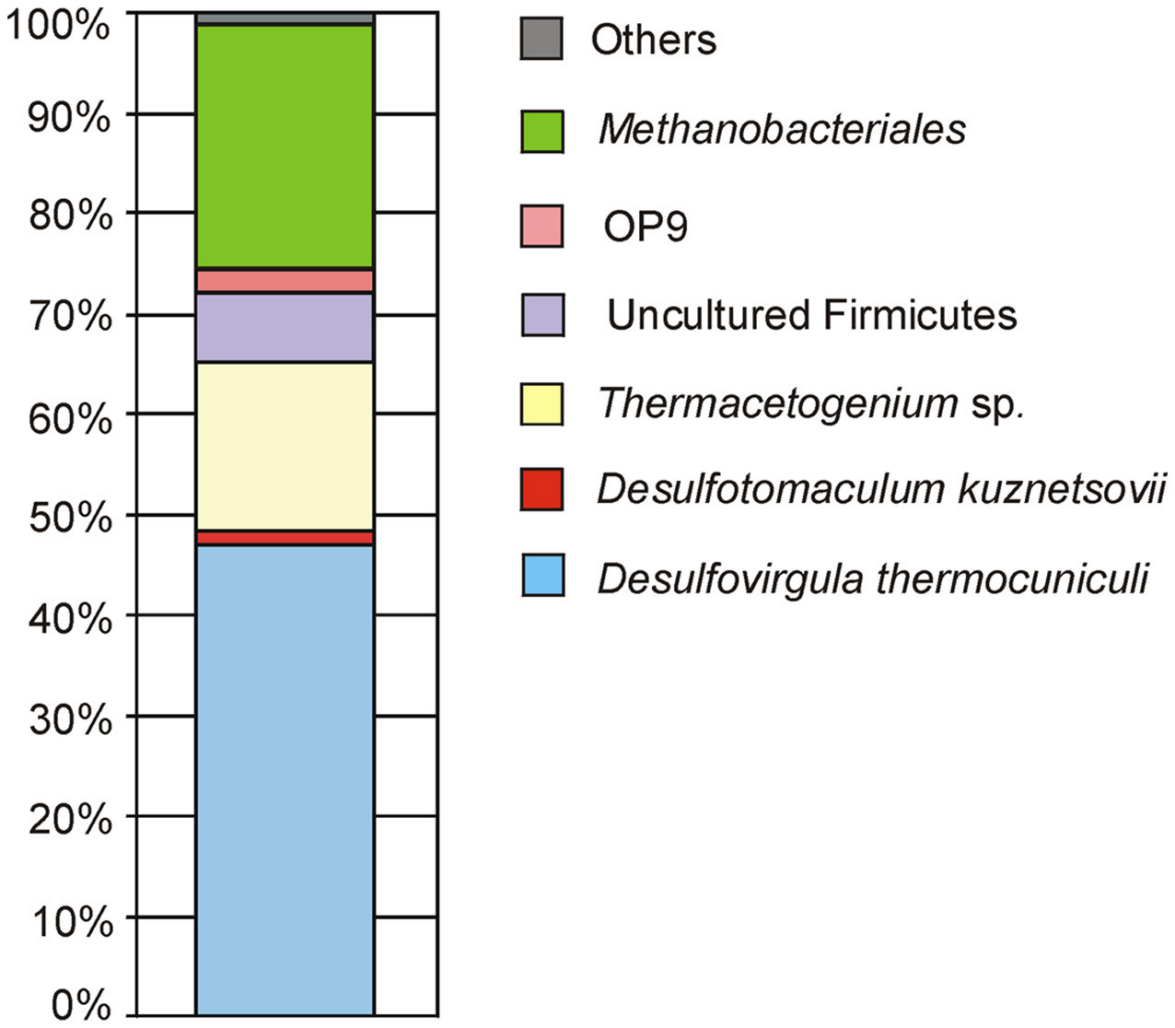
**Composition of 3P well microbial community as characterized by the pyrosequencing of 16S rRNA gene variable fragments**.

Pyrosequencing analysis of 16S rRNA gene fragments was performed for the total DNA isolated from the borehole water sample of June 2011. Bacteria accounted for 76% of all 16S rRNA reads and were mostly represented by *Firmicutes* (Figure 2). Bacteria closely related to the sulfate-reducing thermophile *Desulfovirgula thermocuniculi* were the dominant component of the community (46.9% of the total). The second most abundant bacterial group (16.7%) comprised several lineages related to the genus *Thermoacetogenium*. Sulfate-reducer *Desulfotomaculum kuznetsovii* (Nazina et al., 1988) accounted for 1.3% of prokaryotes. Other *Firmicutes* of uncultured genera comprised 6.9% of the whole community. Members of the candidate division OP9 (Hugenholtz et al., 1998) accounted for 2.4% of the community. This group includes organotrophic bacteria proposed to ferment polysaccharides (Dodsworth et al., 2013). Other bacteria were about 1.3% of the total. Among them were *Bellilinea caldifistulae* from the phylum *Chloroflexi* (0.15%) and *Ignavibacterium album* (0.13%) from the phylum *Ignavibacteriae*.

Archaeal sequences comprised about 24% of all 16S rRNA gene fragments. Most of them could be assigned to the hydrogenotrophic *Methanothermobacter thermautotrophicus* frequently found in aquifers. Among the other archaeal 16S rRNA sequences, members of the genera *Methanobacterium* and *Methanosaeta* were identified in minor amounts (<0.3%).

Overall, the microbial community diversity and richness, characterized by Shannon and equitability indices, is rather low (Table 4) in comparison with values reported for subsurface sediments and oil reservoirs, where Shannon indexes from 2 to 4 are typically reported (for example, Biddle et al., 2011; Gao et al., 2016). This is especially evident for the archaeal part of the community characterized by the presence of just a few species. Thus, in the pyrosequenced DNA sample, the two groups represented by the highest number of sequences—*Desulfovirgula* and *Methanothermobacter* spp.—were those consistently detected by DGGE as the components of the deep subsurface prokaryotic community.

**Table 4:** **Observed bacterial and archaeal diversity estimates based on 97 and 95% OTU levels**.

	**Observed OTUs**		**Shannon's index**		**Equitability**	
	**97%**	**95%**	**97%**	**95%**	**97%**	**95%**
Bacteria	451	305	1.95	1.90	0.32	0.33
Archaea	17	7	0.13	0.11	0.05	0.06

### PCR-DGGE analysis of bacterial diversity in microbial mats

Microbial mats on the wooden conduit construction on the outflow pathway of the borehole water were about 1–2 mm thick and gelatinous and had a colorless, light pink, or pink-grayish hue (Figure S1). Based on the PCR-DGGE results (Table 3), completely different bacteria dominated mat communities in the two samplings in August 2009 and February 2010. Various

*Bacteroidetes* (*Flavobacterium* and *Hydrogenophaga* spp.) were present in August 2009 (colorless and light pink mats), while *Hydrogenophilus* spp. and uncultured *Deferribacteres* were detected in February 2010 (mainly grayish mats).

### Batch and continuous enrichments from borehole water and microbial mats

Batch enrichment cultures were obtained on the media, containing diverse organic substrates in either the presence or absence of external electron acceptors, which were inoculated with borehole water samples (Table 5). PCR-DGGE analyses of the enrichment cultures showed the presence of phylogenetically diverse prokaryotes, most of them belonging to Bacteria.

**Table 5:** **Prokaryotic diversity in enrichment cultures obtained from 3P samples according to PCR-DGGE analyses of 16S rRNA genes**.

**Source, date**	**Designation**	**Substrate, acceptor, temperature**	**Closest cultured/uncultured relative, % of 16S rRNA identity**
Water, Aug 2009	3Pe-A2009-1bac	Microcrystalline cellulose, ${\text{SO}}_{4}^{2}$, 47°C	*Thermoanaerobacterium thermosaccharolyticum*, 90%
	3Pe-A2009-2bac		*Bellilinea caldifistula*e, 99%
	3Pe-A2009-3bac		*Sulfurihydrogenibium azorense*, 89%
	3Pe-A2009-4bac	Cellobiose, ${\text{SO}}_{4}^{2-}$, 60°C	*Desulfotomaculum thermosubterraneum*, 99%
Water, Feb 2010	3Pe-F2010-1bac	Acetate, O_2_ (4,5 %), 50°C^a^	*Hydrogenophilus thermoluteolus*, 99%
	3Pe-F2010-2bac	Acetate, O_2_ (4,5 %), 70°C^a^	
	3Pe-F2010-3bac		
Water, March 2010	3Pe-M2010-1bac	Lactate, 50°C	*Gracilibacter thermotolerans*, 90%
	3Pe-M2010-2bac		*Proteiniphilum acetatigenes*, 93%
	3Pe-M2010-3bac	Lactate, 70°C	*Caloribacterium cisternae*, 97%
	3Pe-M2010-4bac		
	3Pe-M2010-5bac		Candidate division OP9 bacterium SCG 091030-14, 98%
Mat, March 2010	3Pe-M2010-6bac	Lactate, Fe(III), 50°C	*Hydrogenophilus thermoluteolus*, 99%
	3Pe-M2010-7bac		*Syntrophobotulus glycolicus*, 97%
	3Pe-M2010-8bac	Peptone, Fe(III), 70°C	*Brassicibacter mesophilus*, 99%
Water, July 2012	3Pe-J2012-1bac	H_2_ (100 %), As(V), 54°C	*Thauera butanivorans*, 98%
	3Pe-J2012-2bac	Glucose+yeast extract, O_2_, 54°C	*Thauera butanivorans*, 98%
	3Pe-J2012-3bac	Microcrystalline cellulose, 50°C	*Melioribacter roseus*, 100%
Water, Sept 2012	3Pe-S2012-1bac	Gelatine, ${\text{SO}}_{4}^{2-}$, 50°C	*Delftia tsuruhatensis*, 99%
	3Pe-S2012-2bac		*Desulfovirgula thermocuniculi*, 99%
	3Pe-S2012-3bac		*Calditerrivibrio nitroreducens*, 90%
	3Pe-S2012-4bac		*Deferribacter desulfuricans*, 90%
	3Pe-S2012-1arc		*Methanothermobacter thermautotrophicus*, 100%
Mat, Aug 2009	NR_074796.1	Microcrystalline cellulose^b^, SO_4_, 47°C	*Melioribacter roseus* gen. nov., sp. nov.
	JQ292916.1		*Ornatilinea apprima* gen. nov., sp. nov.
	KF931641.1		*Mobilitalea sibirica* gen. nov., sp. nov.

a *Continuous culture*.

b *The components of this enrichment represent novel taxa (Podosokorskaya et al., 2013a,b, 2014)*.

Phylum *Firmicutes* was represented by numerous phylotypes, including both cultured and uncultured members of this group. Among other phylotypes, *Bellilinea caldifistulae* (99% similarity) and *Melioribacter roseus* (100% similarity with the type strain; Podosokorskaya et al., 2013a), representing *Chloroflexi* and *Ignavibacteriae* phyla, respectively, should be mentioned as well as *Hydrogenophilus thermoluteolous* (99% identity), a member of *Betaproteobacteria* and a representative of the candidate division OP9 (98% similarity) (Hugenholtz et al., 1998).

Several enrichment cultures were obtained in the presence of insoluble Fe(III) forms and As(V) as the electron acceptors.

The only enrichment culture that contained prevailing phylotypes of the consortium, which has also been identified directly by PCR-DGGE and pyrosequencing of the borehole water, was that incubated at 50°C with gelatin and sulfate. It comprised of *Desulfovirgula thermocuniculi* (99% similarity) and *Methanothermobacter thermautotrophicus* (100% similarity), accompanied by *Delftia tsuruhatensis* (99% similarity) and two uncultured *Deferribacteres*.

The enrichment culture obtained from a 3P microbial mat sample on the medium with microcrystalline cellulose contained three novel genera of hydrolytic bacteria that were subsequently isolated and described as *Melioribacter roseus* gen. nov., sp. nov., *Ornatilinea apprima* gen. nov., sp. nov., and *Mobilitalea sibirica* gen. nov., sp. nov. (Podosokorskaya et al., 2013a,b, 2014). They were able to grow on various polysaccharides, including cellulose, as well as on sugars and peptides.

Two continuous enrichments from the borehole water developed in the bioreactor under microaerobic conditions (4% O_2_). The enrichments were obtained with an incremental temperature increase from 50 to 70°C, with acetate as the sole carbon source and electron donor. The cell density in the bioreactor started at 1 × 10^4^ cells ml^−1^ at 50°C and reached 3 × 10^7^ cells ml^−1^ after 144 h of batch cultivation, thus indicating the beginning of the exponential growth phase. The exponential phase was sustained for 50 h of continuous cultivation under a perfect mixing regimen. Subsequently, the cultivation mode was switched back to batch culture, and the cell density reached its maximum of 7 × 10^9^ cells ml^−1^ within 96 h. The high cell density was accompanied by the formation of biofilms on the interior glass walls, rotating impellers of the bioreactor, and the glass beads used to model the wallrock. When the cells started to lyse, the temperature was increased to 70°C, and the bioreactor was switched again to continuous mode. The growth ensued for about 100 h but to a lesser cell density (2·10^8^ cells ml^−1^). Acetate consumption correlated well with the growth curve of the culture (Figure S2). DGGE analysis throughout the enrichments at 50 and 70°C showed only one phylotype, which is a close relative of *Hydrogenophylus thermoluteolus* (99% similarity).

## Discussion

The oil exploration well 3P near Parabel' in Tomsk Region was drilled to the recorded depth of 2775 m 60 years ago and has never been an oil production well. For this study, the continuous outflow of deep saline thermal water provided an easy and reliable access to the deep subsurface biosphere. Assuming an annual average surface temperature close to 0°C, the 50°C temperature of water emerging from the borehole is compatible or slightly lower than calculated according to the typical geothermal gradient of 2–3°C/100 m (Banks, 2012; Slobodkin and Slobodkina, 2014). Considering the fact that the water has potentially cooled during passage up the borehole, it seems likely that the majority of the overflowing water is derived from close to the base of the borehole. Analyses of water performed three times during one year (2009–2010) showed that the borehole water is neutral and highly reduced, with salinity around 1.4% and a moderate concentration of dissolved organic carbon. The Na content of 4.1–4.3 g l^−1^ and the Cl^−^ content of 7.5–8.6 g l^−1^ (2013 data) suggest that the water could represent a mixture of fresh meteoric recharge water with around 40% marine water, which could be original connate marine sedimentary water or be derived from a subsequent marine inundation of the terrain. Extremely low Mg/Sr mass ratios (~ 0.06) suggest that Sr has accumulated strongly during a prolonged residence time, while Mg has been depleted, possibly by dolomitization. Water temperature and chemical composition were not completely constant. These changes could not be explained as seasonal, as the samples from February and August 2010 were more similar than those of August 2009 and 2010. One hypothesis is that atmospheric pressure or tidal effects may affect the flow rate and, thus, the temperature and the dissolved gases (the slower the upflow rate in the borehole, the more heat is lost to the surrounding rocks). The possibility of mixing with variable fractions of shallow groundwater (e.g., via leaks in the borehole casing) also exists. However, our previous data show that stable isotope composition of the borehole water, when plotted on an ^18^O vs. ^2^H plot, falls to the right of the global meteoric water line (GMWL). This suggests that the water may partly be derived from meteoric recharge but with the isotopic signature, modified by ^18^O exchange with the aquifer matrix in a mildly geothermal environment, i.e., with a greater component of connate marine water in the 3P well (Banks et al., 2014).

The 5 years monitoring the same site with the same molecular technique reliably demonstrates that the groundwater planktonic microbial community consists of permanent major part and variable minor components. The revealed constant components are sulfate-reducers of the genus *Desulfovirgula* and methanogens of the family *Methanobacteraceae*. Pyrosequencing of 16S rRNA gene fragments showed that these two groups of prokaryotes comprise the majority of sequences (71% of the total amount) in the samples. This fact is in good correlation with repeated PCR-DGGE results. Regarding bacterial part of the community, these results are in contrast with some studies of deep subsurface bedrock-related terrestrial environments where the representatives of *Comamonadaceae* and *Acholeplasmataceae* (Nyyssönen et al., 2014) or *Alicyclobacillaceae* (Miettinen et al., 2015) dominated. However the representatives of the family *Methanobacteraceae* are fairly common for deep subsurface microbial communities in oil and gas bearing environments formed in sedimentary sequences (Ng et al., 1989; Nazina et al., 1995, 2006; Bonch-Osmolovskaya et al., 2003; Mochimaru et al., 2007; Yamane et al., 2011; Frank et al., 2016).

The minor components of the studied community appeared to be highly variable. It is plausible that some of the variability in the community composition could be related to variations in the water chemistry. PCR-DGGE analyses of the August 2009 water samples showed the highest diversity of bacteria. The initial presence of the representatives of the genus *Delftia* has not been observed in subsequent samples. *Delftia* spp. are mesophilic, organotrophic facultative anaerobes capable of degrading organic substrates, including hydrocarbons via aerobic or nitrate respiration. The presence of *Delftia* spp. corresponds to the lowest temperature, the highest ammonia concentration, and significant amounts of organics in August 2009 (Table 1). It was recently found that *Delftia* isolates originating from a deep subsurface aquifer are able to grow anaerobically with insoluble electron acceptors mimicked by electrodes (Jangir et al., 2016). It could be assumed that bacteria of this genus are present in the attached part of the microbial community growing organotrophically with minerals as the electron acceptors and are spread in the water at favorable conditions, as in August 2009, when an easily accessible electron acceptor nitrate was abundant. Noteworthily, sequences related to *Delftia* comprised the majority of RNA library from a borehole in Pyhäsalmi mine (Finland) representing the main active part of the microbial community (Miettinen et al., 2015). Similar sequences have been recovered from saline hydrothermal water in a Mexican mine (Ragon et al., 2013). Another minor component of the 3P well community—bacteria of the genus *Desulfotomaculum*—appeared in August 2009, both in water samples and in the enrichment culture with cellobiose and sulfate; they were also detected in June 2011 by 16S rRNA gene fragments pyrosequencing analysis. Their presence in the water could be attributed to the elevated concentration of sulfate (4.2 mg l^−1^ in August 2009 in comparison with that of <2.0 mg l^−1^ in February and August 2010). However, most of the detected *Firmicutes* had a very low level of 16S rRNA gene similarity with cultured taxa (83–89%; Table 3). Thus, their presence is unlikely to be directly connected with the water characteristics and chemical composition.

Both the planktonic community and the batch enrichment cultures contained many previously uncultured prokaryotes that belong in majority to the phylum *Firmicutes*. Many authors consider *Firmicutes* an important component of subsurface microbial communities (Davidson et al., 2011; Itävaara et al., 2011; Miettinen et al., 2015; Frank et al., 2016). Enrichments with microcrystalline cellulose and sulfate at 47°C contained three types of anaerobic organisms: (i) unidentified *Clostridia* (90% similarity with *Thermoanaerobacterium thermosaccharolyticum*), (ii) unidentified *Aquificae* (89% similarity with *Sulfurihydrogenibium azorense*), and (iii) *Bellilinea caldifistulae* (99%). Many *Clostridia* are known to hydrolyze cellulose and ferment glucose to volatile fatty acids. *B. caldifistulae* can utilize some short chain volatile fatty acids and a range of carbohydrates and was also identified in the planktonic community by pyrosequencing.

Another culture under comparable enrichment conditions but from microbial mat samples yielded three hydrolytic bacteria that were subsequently isolated in pure cultures and described as (i) *Melioribacter roseus* gen. nov., sp. nov., (ii) *Ornatilinea apprima* gen. nov., sp. nov., and (iii) *Mobilitalea sibirica* gen. nov., sp. nov. (Podosokorskaya et al., 2013a,b, 2014). Their combined spectrum of substrates includes cellulose as well as various di- and polysaccharides and proteinaceous substrates. *M. roseus* is a facultative anaerobe and can use arsenate, insoluble Fe(III) oxide ferrihydrite, and nitrite as external electron acceptors. This bacterium was also found in the enrichment culture from a borehole water sample (Table 5). It belongs to the new phylum *Ignavibacteriae* (Podosokorskaya et al., 2013a); another representative of which was also detected by PCR-DGGE analysis in the borehole water sample of March 2010 (Table 3). Thus, *M. roseus* and two other polysaccharide-degrading isolates represent, most probably, immobilized organotrophic bacteria that perform anaerobic degradation of buried organic matter in the 3P well community. Moreover, *M. roseus* is capable of oxygen respiration, and, being brought to the surface with the borehole outflow, it could proliferate in periodically aerated microbial mats fed with organic-rich thermal water.

The gelatin-utilizing enrichment culture contained all components of the planktonic prokaryotic consortium: *Delftia, Desulfovirgula* and *Methanothermobacter* spp., together with uncultured representatives of *Deferribacteres*. This suggests that gelatin may successfully substitute for natural polymeric substrates for cultural recoveries of prokaryotes from the subsurface.

Fe(III), in the form of glauconite (a mica group phyllosilicate), was added as an external electron acceptor in some enrichment cultures. It is an insoluble electron acceptor candidate in geothermal systems (others include ferrihydrite, Fe(III)-oxyhydroxides, and iron-containing aluminosilicates), as it is widely present in marine sedimentary depositional sequences of the deep West Siberian basin (Nikitenko, 2009). Previous studies have demonstrated a broad ability for ferric iron reduction of microorganisms inhabiting the deep subsurface biosphere and oil wells in particular (Slobodkin et al., 1999; Li et al., 2006). Direct enrichments using glauconite as an electron acceptor were obtained only from the mat samples and contained bacteria of *Brassicibacter, Hydrogenophilus*, and *Syntrophobotulus* genera. Representatives of these taxa have not been previously shown to reduce Fe(III), although *Hydrogenophilus* can grow chemolithoautotrophically with molecular H_2_ as the electron donor, CO_2_ as the carbon source, and oxygen or nitrate as external electron acceptors. As the reduction of nitrate may involve multiheme *c*-type cytochromes, which are also the major determinants of Fe(III) reduction (Sharma et al., 2010), one might expect the iron-reducing activity in a *Hydrogenophilus* strain enriched with glauconite in our experiments.

Two major components of the planktonic community in the borehole water were consistently present: sulfate-reducers assigned to *Desulfovirgula thermocuniculi* and methanogens of the genus *Methanothermobacter*. Thermophilic sulfate-reducing bacteria in oil formation water have been documented in many studies (e.g., Magot et al., 2000; Slobodkin and Slobodkina, 2014) and are especially characteristic for submarine oil deposits where sulfates are easily available. Thermophilic sulfate-reducers of the genera *Desulfotomaculum, Desulfacinum, Thermodesulfobacterium*, and *Thermodesulforhabdus*, as well as hyperthermophilic archaea of the genus *Archaeoglobus*, have been either isolated or detected by molecular methods in oil wells of the North Sea (Beeder et al., 1994; Rees et al., 1995; Nilsen et al., 1996). In terrestrial high-temperature oil reservoirs where the concentration of sulfates is much lower, sulfate-reducers or their activity have also been detected (Nazina et al., 1995, 2006; Bonch-Osmolovskaya et al., 2003; Frank et al., 2016). The most commonly mentioned sulfate-reducers in high-temperature oil reservoirs are thermophilic members of the genus *Desulfotomaculum*. However, quantitative data on their presence in microbial communities of organic-rich deep subsurface habitats have not been presented to date. In this study, sulfate-reducing *Desulfotomaculum* spp. was only detected in borehole water as a minor component. Another sulfate-reducing Firmicute—*Desulfovirgula thermocuniculi—*was found to be a key constituent of the planktonic microbial community of the borehole water. The type strain of *D. thermocuniculi* was isolated from a geothermally influenced underground mine sample in Japan (Kaksonen et al., 2007). *D. thermocuniculi* is able to oxidize H_2_ and organic acids with sulfate as the electron acceptor or perform organic acid fermentation. Considering the extremely low concentration of sulfates in the borehole water (from <2 to 4.23 mg l^−1^), we assume that in the borehole community *D. thermocuniculi* grows by the oxidation of carboxylic acids present in the water, and the produced hydrogen is scavenged by hydrogenotrophic methanogens via interspecies hydrogen transfer, which takes place at the low availability of sulfates.

Methanogens of the family *Methanobacteraceae* are common members of deep subsurface microbial communities, especially of those associated with oil deposits (Ng et al., 1989; Nazina et al., 1995, 2006; Bonch-Osmolovskaya et al., 2003; Mochimaru et al., 2007; Yamane et al., 2011; Frank et al., 2016). *Methanobacteraceae* were found to be consistent components of the borehole water. According to pyrosequencing analysis, *Methanobacteraceae* comprised 24% of the total amount of sequences. In contrast, representatives of *Methanosaeta* were only detected in 2011 by 16S rRNA pyrosequencing and in only minor quantities. *Methanosarcinales* were not detected in the borehole water, while these methanogens were reported to dominate a high-temperature oil reservoir in California (Orphan et al., 2003).

A syntrophic mechanism of acetate conversion to methane occurring in high-temperature oil reservoirs has been proposed by Nazina et al. (2006), whereby *Thermoacetogenium phaeum* (Hattori et al., 2000) degrades acetate to hydrogen and CO_2_, and *Methanothermobacter* spp. converts these products to CH_4_. The presence of bacteria related to *T. phaeum* in the formation water of oil reservoirs has been previously noted in Japan (Yamane et al., 2011). *Firmicutes* related to *T. phaeum* were detected in the 3P borehole water by PCR-DGGE in 2009 and by pyrosequencing in 2011; in the latter sampling, they represented a significant part (16%) of the microbial community. Thus, acetate-utilizing syntrophs may participate in methanogenesis in the borehole microbial community. In the absence of *T. phaeum*, syntrophy with methanogens may involve other members of uncultured *Firmicutes* detected in five borehole water samples.

Another point is that the prevalence of sulfate-reducers and methanogens may indicate reverse methanogenesis by an anaerobic, methane-oxidizing consortium, with sulfate as the electron acceptor (Knittel and Boetius, 2009). The reversibility of methyl-coenzyme M reductase, the key enzyme of methanogenesis, is supported by thermodynamic and kinetic considerations (Thauer, 2011) and by the activity assays with purified enzymes from *Methanothermobacter marburgensis* (Scheller et al., 2010) and *M. thermautotrophicus* (Chen et al., 2012). The thermogenic nature of methane in the borehole water, as indicated by the carbon isotopic composition, suggests conditions favorable for methanotrophy coupled to sulfate reduction rather than methanogenesis.

When the reduced water containing energy-rich substrates, but lacking easily available electron acceptors, flows out of the well, microbial mats develop on the wooden conduit along the flow pathway of the borehole water. These were found to consist of either *Hydrogenophilus thermoluteolus* and uncultured *Deferribacteres* or *Flavobacterium* and *Hydrogenophaga*-related organisms (Table 3). None of these prokaryotes were detected in the borehole water by PCR-DGGE or pyrosequencing. *H. thermoluteolus* was present in lactate-utilizing enrichment cultures amended with glauconite from the microbial mat, and it appeared to be the only phylotype in the continuous-flow microaerophilic enrichment obtained from the borehole water. This continuous enrichment may be regarded as a laboratory model of a microbial mat fed with the thermal borehole water, which brings *H. thermoluteolus* from its subsurface habitats. The ability of the enriched *H. thermoluteolus* to form thick biofilms, even at turbulent mixing, indicates that this microorganism could thrive at intermediate layers of the borehole, where oxygen, putatively released from extrinsic shallow groundwater, is mixed with intensive connate hot borehole outflow. While *H. thermoluteolus* could be a minor component in the borehole water community, it becomes dominant in the microbial mats as a result of more favorable conditions.

It should be emphasized that the deep subsurface, we were able to access via borehole water sampling, has two (planktonic and immobilized) microbial components that are both supported by organic matter contained in the Mesozoic sedimentary deposits. Biopolymers from that source could be degraded by anaerobes able to attach to various surfaces and possessing multiple hydrolytic catabolic pathways, such as the representatives of the phylum *Ignavibacteriae* (Podosokorskaya et al., 2013a). Aqueous organic solutes produced by hydrolytic microorganisms can potentially be completely oxidized by sulfate-reducers in the planktonic community (e.g., *Desulfovirgula* and *Desulfotomaculum* spp.) or by syntrophic methanogenic associations, such as sulfate-reducers or *Thermacetogenium* and *Methanothermobacter* spp. Another option could be reverse methanogenesis in syntrophy with sulfate-reducers, recycling methane to the biomass or yielding CO_2_ and H_2_S. However, considering the thermogenic nature of methane (i.e., the mixture of biogenic and abiogenic CH_4_) in the borehole, neither methanogenesis nor methanotrophy prevalence can be reliably inferred from our data. At the artesian outflow of the borehole, aerobic or microaerobic processes can drive the oxidation of soluble organics supporting the growth of microbial mats formed by minor or immobilized components of the subsurface water ecosystem. The presence of many “uncultured” microorganisms in enrichment cultures provides hope for their successful cultivation and, thus, to the understanding of their metabolic function in the deep subsurface environment.

## Author contributions

YF, VK, SG, DB, AG, OP, and OK contributed to field work. YF, AG, OP, and SG obtained enrichment and pure cultures. YF, DB, and OK contributed to physical-chemical analysis. YF, AG, NC, AYM, and OK performed PCR-DGGE and phylogenetic analysis. VK, AVM, and NR performed pyrosequencing analysis. EB, OK, SG, and YF designed the work. EB, OK, SG, YF, NR, DB, AYM, and OP wrote the manuscript.

## Funding

This work was supported by the Russian Science Foundation [14–24–00165] (studies on biogeochemical cycling of C1-compounds), Russian Federation Agency of Science and Innovations (field work), and Russian Foundation for Basic Research [13–04–40205]. Analysis of microbial communities by pyrosequencing of 16S rRNA genes was supported by the Russian Science Foundation [14–14–01016] to NR group. Molecular screening of sulfate-reducing enrichments was performed with support of the Russian Science Foundation [14–14–00427].

### Conflict of interest statement

The authors declare that the research was conducted in the absence of any commercial or financial relationships that could be construed as a potential conflict of interest.
