# Supplementary material for: Stable and Variable Parts of Microbial Community in Siberian Deep Subsurface Thermal Aquifer System Revealed in a Long-Term Monitoring Study
Source: Front Microbiol. 2016 Dec 27;7:2101. doi: 10.3389/fmicb.2016.02101 (PMC5187383; doi:10.3389/fmicb.2016.02101)
Supplement: Supplementary file 1 [file DataSheet1.PDF]

Stable and Variable Parts of Microbial Community in Siberian Deep Subsurface Thermal Aquifer System Revealed in a Long-Term Monitoring Study

Yulia A. Frank, Vitaly V. Kadnikov, Sergey N. Gavrilov, David Banks, Anna L. Gerasimchuk, Olga A. Podosokorskaya, Alexander Y. Merkel, Nikolai A. Chernyh, Andrey V. Mardanov, Nikolai V. Ravin, Olga V. Karnachuk and Elizaveta A. Bonch-Osmolovskaya

Supplementary material

Table S1. Key inorganic composition (in mg l^{-1}) of the borehole water sampled in August 2013.

**Cations and metals**		**Anions**	
Ca	1166	Cl^-	8562
Mg	5.4	SO_4^{2-}	<25
Na	4339	NO_3^-	<15
K	37.4	Br^-	32.7
Fe	0.078	F^-	<2.5
Mn	0.348		
Sr	86.4	Alkalinity	3.0 meq/l
Ba	16.8		
Li	0.66		
**Other components**			
Si	19.8		
As	0.126		
B	10.2		

Table S2. Oligonucleotide primers and PCR conditions used for amplification.

Primer pair	Target gene	Sequence (5'→3')	Temperature cycling	Components added				Reference
				$MgCl_2$ (mM)	dNTP (µM)	Taq (U)	Primers (µM)	
27F-1492R	16S rRNA of *Bacteria*	27F: AGA GTT TGA TCC TGG CTC AG<br>1492R: GGT TAC CTT GTT ACG ACT T	Initial denaturation 95 ºC, 20 s<br>6 cycles: 95 ºC, 10 s, 45 ºC, 20 s, 72 ºC, 90 s<br>30 cycles: 95 ºC, 10 s, 55 ºC, 20 s, 72 ºC, 1.5 min<br>Final elongation 72 ºC, 3 min	2.5	100	1.25	0.2	DeLong (1992)<br>Weisburg *et al*. (1991)
GC-BacV3f*-907R	16S rRNA of *Bacteria*	BacV3f: CCT ACG GGA GGC AGC AG<br>907R: CCG TCA ATT CMT TTG AGT TT	Initial denaturation 95 ºC, 5 min<br>30 cycles: 95 ºC, 20 s, 50 ºC, 1 min, 72 ºC, 1.5 min<br>Final elongation 72 ºC, 10 min	2.5	100	1.25	0.2	Muyzer *et al*. (1996)<br>Lane (1991)
21F-958R	16S rRNA of *Archaea*	21F: TTC CGG TTG ATC CYG CCG GA<br>958R: YCC GGC GTT GAM TCC AAT T	Initial denaturation 95 ºC, 5 min<br>30 cycles: 94 °C, 45 s, 55 °C, 1 min, 72 °C, 1.5 min<br>Final elongation 72 ºC, 10 min	1.5	200	1.25	0.5	DeLong (1992)
Parch519f-Arch915R*	16S rRNA of *Archaea*	Arch915R: GTG CTC CCC CGC CAA TTC CT<br>Parch519f : CAG CCG CCG CGG TAA	Initial denaturation 96 ºC, 4 min<br>35 cycles: 94 °C, 30 s, 57 °C, 40 s, 72 °C, 40 s<br>Final elongation 72 ºC, 10 min	1.5	200	1.25	0.5	Coolen *et al*. (2004)

*- A GC-clamp 5’-CGC CCG CCG CGC CCC GCG CCC GGC CCG CCG CCC CCG CCC C-3’ (Muyzer *et al*., 1993) was attached to the 5’-end of primers

Table S3. Composition of trace element stock solutions A (acidic) and B (basic) used for continuous enrichment cultures.

**Component**	**Concentration in the stock solution (mM)**
Solution A (x100)	
$CrCl_3$	0.013
$AlCl_3 \cdot 6H_2O$	0.79
KBr	40.2
KI	0.79
$MnCl_2 \cdot 4H_2O$	0.65
$FeCl_2$	10.3
$CoCl_2 \cdot 6H_2O$	0.005
$NiCl_2 \cdot 6H_2O$	0.065
$CuCl_2$	0.009
$ZnCl_2$	0.22
$BaCl_2 \cdot 2H_2O$	10.4
As_2O_3	0.19
$NaVO_3 \cdot 2H_2O$	0.093
H_3BO_3	59.1
HCl	100
Solution B (x1000)	
$LiOH \cdot H_2O$	96.3
$Na_2SiO_4 \cdot 9H_2O$	547.0
$Na_2WO_4 \cdot 2H_2O$	0.006
$Na_2SeO_3 \cdot 5H_2O$	1.91

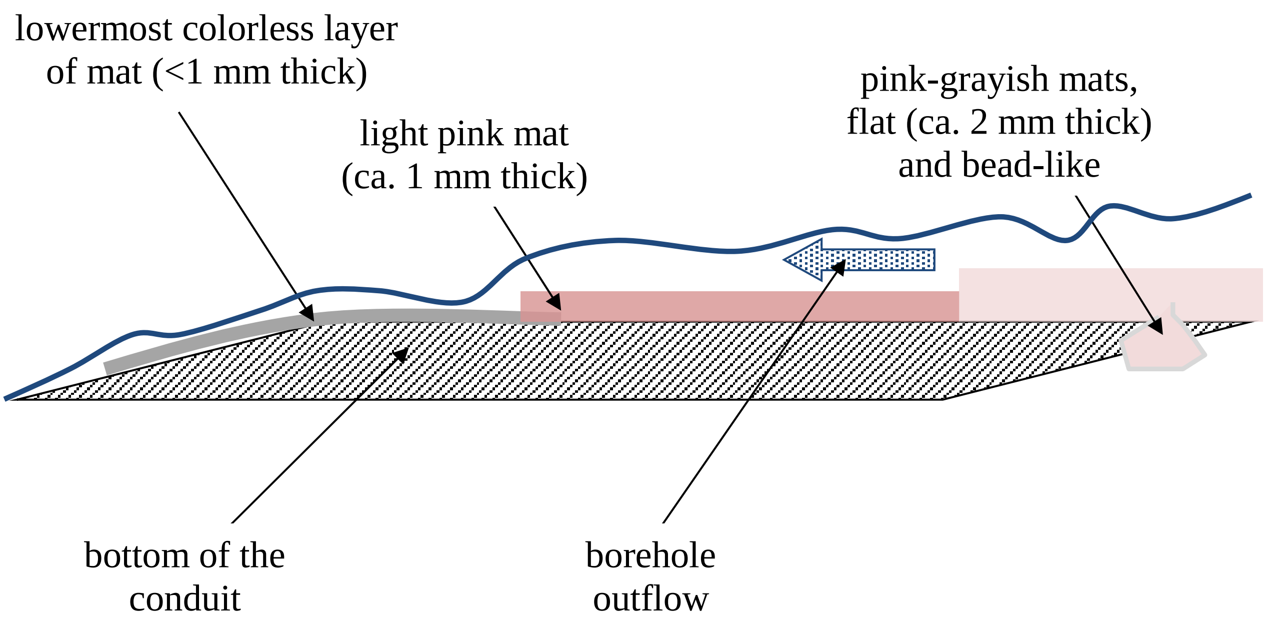

Fig. S1. Schematic representation of microbial mats on the surface of the wooden conduit.

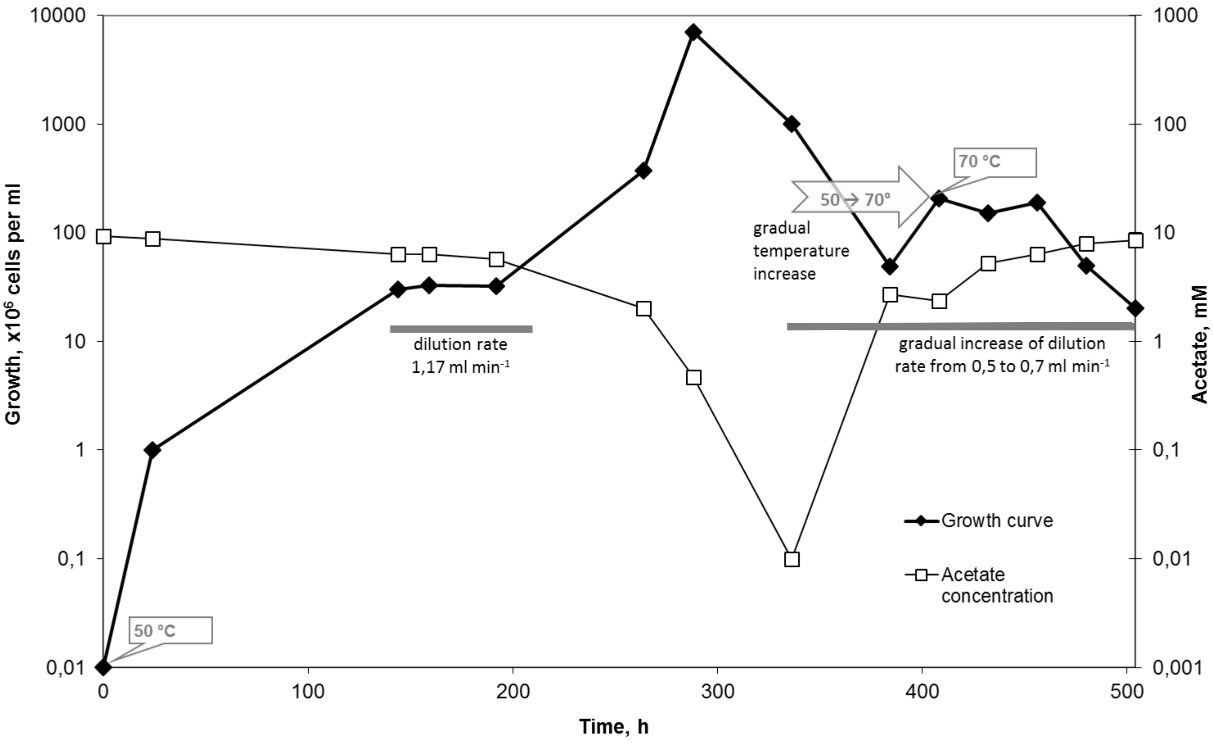

Fig. S2. Growth and acetate consumption of continuous enrichment culture obtained from water sample taken on February 2010.

Note logarithmic scales.

Markers on both curves mask the error bars.

Indicated changes of cultivation temperature and dilution rate are bound to the timeline. Bold gray straight lines highlight periods of continuous cultivation; beyond these periods, the enrichment was cultivated in batch mode. The dilution rate was calculated and gradually adjusted according to the growth rate.
